# The sex pheromone of a globally invasive honey bee predator, the Asian eusocial hornet, *Vespa velutina*

**DOI:** 10.1038/s41598-017-13509-7

**Published:** 2017-10-11

**Authors:** Ping Wen, Ya-Nan Cheng, Shi-Hao Dong, Zheng-Wei Wang, Ken Tan, James C. Nieh

**Affiliations:** 1 Key Laboratory of Tropical Forest Ecology, Xishuangbanna Tropical Botanical Garden, Chinese Academy of Sciences, Kunming, Yunnan Province 650223 China; 2 University of Chinese Academy of Sciences, Beijing, 100049 China; 3 Eastern Bee Research Institute, Yunnan Agricultural University, 650201 Kunming, China; 4 Division of Biological Sciences, Section of Ecology, Behaviour, and Evolution, University of California, San Diego, La Jolla, California, USA

## Abstract

The Asian hornet, *Vespa velutina*, is an invasive, globally-distributed predator of European honey bees and other insects. To better under its reproductive biology and to find a specific, effective, and low-impact control method for this species, we identified and tested the key compounds in *V*. *velutina* sex pheromone. Virgin gynes (reproductive females) produced this sex pheromone in the sixth intersegmental sternal glands of their abdomens. The active compounds were 4-oxo-octanoic acid (4-OOA, 10.4 μg bee^−1^) and 4-oxo-decanoic acid (4-ODA, 13.3 μg bee^−1^) at a 0.78 ratio of 4-OOA/4-ODA. We synthesized these compounds and showed that male antennae were highly sensitive to them. Moreover, males were only strongly attracted to a 4-OOA/4-ODA blend at the natural ratio produced by gynes. These results provide the first demonstration of an effective way to lure *V*. *velutina* males, and the first chemical identification of a sex pheromone in the eusocial hornets.

## Introduction

*Vespa velutina* is a widespread global predator of honey bees that, over the past decade, has invaded Europe and Korea, where it poses problems to honey bees^1^ and to human health^2,3^. Its spread in Europe has been more rapid than in Korea^1^, perhaps because it faces competition from other *Vespa* species in Korea. *Vespa velutina* is strongly attracted to the olfactory signals and cues produced by honey bee colonies^4^, but the defences of the western honey bee, *Apis mellifera*, are relatively weak in comparison to those of *A*. *cerana*, which has co-evolved with this formidable hunter. Hornets are 8-fold more successful at capturing the former as compared to the latter^5^. Multiple European countries, including Turkey and Balkan nations, have been invaded by this predator, and much of western Europe is at risk of invasion^6^. *Vespa velutina* is found in rural and urban areas and also causes human harm and pain, particularly for individuals allergic to hornet stings^2,3^. The European economic impact is high: major colony losses have led some beekeepers to abandon apiculture^6^. *Vespa velutina* is difficult to control because its colonies can rapidly proliferate, it has relatively high nest densities, and it can be challenging to find nests in non-urban areas^7^. A potential solution would be the use of sex pheromone traps, which are an effective way to monitor insect populations, contribute to insect control, are specific, and have low environmental impact because they usually do not use toxins^8^. However, no sex pheromone traps have yet been developed for *V*. *velutina*. In fact, what little is known about *V*. *velutina* mating is largely anecdotal^9,10^.

Mating in social hornets and wasps can occur at nests^11^ or at alternative mating sites^12^. In *Polistes fuscatus*, some males defend territories where females nest and hibernate, while other males patrol where females forage and attempt to copulate with these females and even other flying insects^12^. Males of the European hornet, *Vespa crabro*, have similar strategies. Some patrol nest sites, while others monitor sunny spots in vegetation, up to 185 m away from nests^13^. *Vespa mandarinia* evidently mates only at nest entrances^14^. In other *Vespa* species, mating may take place far from the nest^14^, suggesting the need for long distance signalling.

In eusocial wasps, including social *Polistes*, *Vespa*, and *Vespula*, sexual signalling likely plays a key role in allowing virgin gynes (reproductive females) and males to find each other^15^. Although males generally seek out females, females can be attracted to males, as in *Polistes exclamans* ^16^. Short-distance^17,18^ and long-distance^13^ attraction occurs, and both visual and olfactory cues and signals may be important^19^. However, given the limitations of insect visual acuity, long-distance attraction likely relies upon sex pheromones. Sex pheromones are therefore found in a wide variety of social wasps, including *Polistes*, *Vespa*, *Vespula*, and *Dolichovespula* ^19^ species. *Vespula squamosa* ^20^, *Vespula vulgaris* ^9^ and *Vespa crabro* ^13^ males are attracted to virgin gynes and gyne extracts over distances ranging from a few meters (wind tunnel tests^20^) up to hundreds of meters (open field conditions^9^).

Sex pheromones have independently evolved from multiple glandular sources in insects^21^ such as ants^22^, bees^22^, and termites^23,24^. Bumble bees^25^ and eusocial halictine bees^26^ can produce sex pheromones in their cuticular hydrocarbons. Females of the European hornets *Vespa crabro* and *Polistes dominulus* produce short-distance contact sex pheromones in their antennal glands^17,18^. The hymenopteran sting evolved from an ovipositor, which is only found in females^27^. Thus, it is not surprising that the sting venom gland produces sex pheromone in several polistine wasp species^11,12,16,28,29^. However, the chemical components and glandular sources of sex pheromones in wasps and hornets remain poorly understood, even though such pheromones likely play an important role in sexual selection and should be useful for monitoring and controlling the spread of species that, like *V*. *velutina*, have invaded new ecosystems^7,10^.

In particular, the active compounds in the sex pheromones of eusocial hornets, which can be pests, have not been identified^30^, although there is behavioural evidence for a gyne sex pheromone that attracts males in six Asian *Vespa* species^31^. Male responses are species-specific, suggesting that multiple pheromone components are involved^32^. Although a source of short-distance contact sex pheromones has been identified in *Vespa crabro* ^17^, such short-distance pheromones have limited utility as a sex attractant for pheromone traps. We do not know the glandular source or active components of long-distance sex pheromones in *Vespa*.

*Vespa velutina* reproduction follows an annual cycle. Nests are established by an overwintering foundress during the spring^33^. After 4–5 months, the colony reaches its maximum size and produces a large number of gynes and males from September-December (at our research sites), followed by colony die-off ^33^. In preliminary observations, we observed *V*. *velutina* males that appeared to be strongly attracted over long distances to virgin gynes. We therefore focused on testing the hypothesis that *Vespa velutina* uses sex pheromones. We then sought to identify the source of these pheromones and the active compounds involved. Our goals were to understand better the reproductive signalling biology of eusocial hornets and to provide a potential control strategy because this invasive species causes significant ecological and economic harm.

## Materials and Methods

### Insects and sites

Three large, healthy, and growing *V*. *velutina* colonies were collected and transported from Wuding (Yunnan, China) to our research sites. Each day during the gyne mating season (September-December), we opened the carton nest of each colony and collected newly emerged gynes. Gynes were easily identifiable because they are significantly larger than workers. We maintained gynes in separate cages and fed them with odourless, pure sucrose solution (50% w/w) for 3–15 d before they were used in experiments. Six feral hornet colonies were located at the Kunming Botanical Garden (two colonies), Yunnan Agriculture University (two colonies), and Southwest Biodiversity Research Centre (two colonies, Table S1). In the morning, we observed virgin gynes leaving the nest on sunny days (defined as ≤30% cloud cover in the sky) and heading for open areas at the rim of the nearby forest. Gynes repeatedly flew out into these open areas to attract males and then flew back to a more sheltered location within the trees. The closest nests that we were able to locate were approximately 400 to 1,500 m away.

Swarms of males were attracted to each gyne advertisement site, which we therefore call a “male congregation area” (MCA). We located multiple MCA sites by carefully searching at our field sites and watching for the repeated patrolling of males and for gyne flights. These observations helped to identify the MCA sites. However, because natural mating behaviour was relatively rare, we were able to conduct trials at times when natural gynes were not present at our sites, to avoid conflating natural gyne mating with our bioassays. Sample sizes for all experiments are summarized in Table S1.

### Bioassay

To study male attraction to living gynes, we placed each gyne in a small wood cage (1.5 cm × 4.5 cm × 6.0 cm, with bamboo bars that were 2.5 mm diameter and spaced 5 m apart). This cage allowed the gyne to release its sex pheromone and also permitted males to see the gyne once they approached the cage. We used a metal wire to attach this gyne cage to a selected tree branch at MCA sites in Yunnan Agriculture University or the Southwest Biodiversity Research Centre during the 2016 mating season. As controls, we used a clean, empty cage that was tied to a similar tree branch about 7 m away. We tested 12 different gynes over both MCA sites (see Table S1). During the period when males were attracted to gynes, we recorded the total number of males that landed on each cage and also noted the wind speed, time of a day, air temperature (measured with a Kestrel 4000, Kestrel, US), and sunlight intensity (in kilolux, assessed with a TA8123, TASI, CN). We calculated a daily average for these ambient environmental conditions. Males typically sought to mate with gynes between 9:00 and 15:00.

Approaching males were allowed to make one choice and were then caught with an insect net. We captured each male as soon as it made a choice (on a gyne cage they typically landed for >1 s before capture) and did not reuse any males. To ensure independent choices, we only used males that made choices in the absence of other males in the vicinity. We fed odourless pure sucrose solution (50% w/w) to the gynes each 0.5 h to keep them in good condition.

To determine the sex pheromone source, we monitored gyne emergence on a daily basis from colonies that we maintained and captured virgin gynes on their day of emergence. Virgin gynes were cold anesthetized and dissected into two parts, the head-thorax and the abdomen. These two body parts were separately hung on copper wires in a tree at a MCA. Clean wires with no body parts were used as a blank non-visual control treatment. These three treatments were presented as an equilateral triangle (1.0 m per side) to test male attraction. Since males were only attracted to the abdomen (see Results), the head-thorax treatment served as a visual control. We used nine gynes during the mating season (three gynes per site: two sites at Yunnan Agriculture University, and one site at the Southwest Biodiversity Research Centre, Table S1). Each trial lasted for 1 h. After finding that males were strongly attracted to the abdomen alone (see Results), we used filter paper strips (4 mm by 15 mm) to rub the intersegmental tergal (T) cuticles and the intersegmental sternal (S) cuticles to narrow down the potential glandular sources.

For our gland localization bioassay, we initially tried measuring attraction to filter paper alone, but found that males would approach, but not land, on paper alone. Males evidently required the visual appearance of a hornet. We therefore decided to use dead male hornets (killed by freezing) as visual models. In *V*. *velutina*, gynes and males are quite similar in visual appearance^14^. Males and gynes also produce similar major cuticular hydrocarbon components, although some minor components differ^34^ (Fig. S1). However, males are not attracted to males and do not produce alarm pheromone^35^ (Fig. S2), which could repel approaching males. In other hornet species, males are also not attracted to males^9,11^. In a preliminary experiment, we tested 36 males with no putative female sex pheromones added and found that no males approached these models. We therefore used freshly killed males as models in our bioassays.

To bioassay the activity of sex pheromone samples, we applied 2 µL of the extracted pheromone, synthetic pheromone, or the solvent as a control (pure diethyl ether) to freshly-killed males (models). A paper strip (4mm by 15 mm) with putative pheromones or the control solvent was attached to each model with a no. 0 insect pin. We then positioned this model in the tree canopy of a known MCA on sunny days (≤30% cloud cover, >15 °C, RH 45–60%) in December 2016.

To bioassay the attractiveness of different ratios of identified sex pheromone compounds, we applied the synthetic compounds or the solvent control (in 2 µL of pure diethyl ether to paper strips attached to freshly-killed males (see above) on sunny days (conditions as above).

### Cuticular hydrocarbon and pheromone extraction and analysis

To extract and analyse the cuticular hydrocarbons of workers, males, and gynes, we placed a freshly freeze-killed hornet in a clean glass vial with 0.5 mL of n-hexane for 10 min. This extract was then concentrated to 5 μL with a gentle 30 ml/min nitrogen flow through a 0.5 mm diameter needle. The resulting extract was injected into the Gas Chromatograph (GC, see below) in purged, splitless mode for analysis.

Based upon our bioassays, we identified the sixth intersegmental sternal glands as a potential source of sex pheromone. To determine the chemical composition of this secretion in gynes, we rubbed the sixth intersegmental sternal gland surface and (as a control) non-glandular cuticular surfaces with Solid Phase Micro-Extraction (SPME) blue fibres (65 μm PDMS/DVB, Supelco, CA). We used Gas Chromatography-Mass Spectrometry (GC-MS) chemical identification, GC coupled to Electroantennographic Detection (GC-EAD) bioactivity identification, or GC quantification analyses, as appropriate to our question.

In some insects, sex pheromone production changes over time^36,37^. To determine if such temporal changes also occur in *V*. *velutina*, we incubated virgin gynes in a box and provided an artificial photoperiod of 10 h light /14 h dark, simulating natural conditions during the mating season, at 75% relative humidity and 25 °C. Three to five gynes were harvested each hour from 8:00 to 17:00. We extracted the sex pheromone with the selective acid extraction method described below.

### Chemical analyses

For pheromone analyses, newly emerged virgin gynes were caught from three colonies. GC-MS analysis was performed with an HP 7890A-5975C GC-MS system (Agilent, US) following the GC procedure described by Cheng *et al*.^35^. Pheromone samples, including SPME, and solvent extracts were injected in splitless mode, and synthetic pheromone was analysed in split mode with a split ratio of 1:10, both at 250 °C. A HP-5ms (30 m × 250 μm × 0.25 μm, Agilent, US) column was used with 1 mL/min helium as carrier gas. The oven ramp was set as 50 °C for 2 min and then 5 °C/min to 280 °C for 10 min. The 71 eV electron impact ion source was heated to 230 °C. The MS quadrupole was heated to 150 °C. The scanned mass range was set as m/z 28.5 to 300 with a threshold abundance of 10 to detect the trace molecular ions. For GC-FID quantification, we used an HP 7890B GC (Agilent, US). The injection port was set to splitless mode at 250 °C. A HP-FFAP column (30 m × 320 μm × 0.25 μm, Agilent, US) was used with nitrogen at 2 mL/min as carrier gas. The oven ramp temperature was 180 °C for 2 min then 10 °C/min to 230 °C for 10 min.

### Microscale chemical analyses

To determine the position of the carbonyl group, we selectively extracted the oxo acid pheromone in 250 μL of 0.2 mol/L NaOH. We then added 0.2 mg NaBH_4_. Following this, 30 μL of 1 mol/L H_2_SO_4_ was slowly added (1 μL at time) to the mixture until bubbles were no longer observed. The resulting mixture (containing H_2_SO_4_ and sodium hydroxyl carboxylate) was lactonized at 60 °C, stirred for 1 h, and then extracted using headspace SPME (65 μm blue PDMS/DVB fibre) at 70 °C for 1 h. For MS identification, the diagnostic ion for the lactone ring was used to determine the position of the carbonyl in the pheromone structures. We also compared the derivatives to commercially available, pure standards.

### Chemical standards

Authentic standards of gamma-octalactone and gamma-decanlactone were purchased from TCI (Japan). The 4-oxo-octanoic acid (CAS# 4316-44-3) and 4-oxo-decanoic acid (CAS# 4144-54-1) were respectively synthesized via hydrolysis and oxidation of gamma-lactones using the green organic synthesis method^38^. We hydrolyzed 0.01 mol of each lactone with 20 mL of 1.0 mol/L NaOH under reflux (100 °C) for 3 h. The mixture was then cooled on ice to 0 °C. We prepared a pH 8 NaClO solution at 0 °C (ice bath) by adjusting the pH of 21% NaClO in 1 mol/L NaOH solution via the dropwise addition of 1 mol/L HCl, and buffered the pH by adding 5% NaHCO_3_. A large quantity (30 ml) of this 0 °C, pH 8 NaClO solution was added, drop by drop, to the cooled and hydrolyzed mixture at 0 °C. The mixture was then stirred at 0 °C for 1 h until all alcohols were eliminated. The resulting mixture was then acidified to pH 3 with 1.0 mol/L HCl and extracted three times with 10 ml of DCM. The organic layer was then washed with saturated NaCl solution and dried with anhydrous MgSO_4_. We then evaporated the solvent, and purified the crude product with silica chromatography. The crude product (1.2 g for 4-oxo-octanoic acid and 1.3 g for 4-oxo-decanoic acid) was transferred to the top of a silica column (30 cm in length, 2.5 cm in diameter) and eluted with a mixture of hexane, ethyl acetate, and acetic acid (ratio of 4:1:0.1). The acid fraction was concentrated and evaporated to obtain the pure oxo-acids. We thereby obtained 1.0 g 4-oxo-octanoic acid (>99% purity assayed via GC) and 1.1 g 4-oxo-decanoic acid (>99% purity).

### GC-EAD and EAG analyses

We used a custom GC-EAD and EAG system^35,39^, to test male antennal electrophysiological responses. The response of this system was significantly improved with a highly sensitive LMP7721 operational amplifier (Texas Instrument, USA). Antennae of males collected from multiple different MCA sites were used (sample sizes in Table S1). We generated dose-response curves by tested each antenna with multiple doses of the same compound. To test each compound, we used one antenna from a different hornet. We used an increasing concentration series, adding 1 µL of the test compounds (1 ng/µL, 10 ng/µL, 100 ng/µL, 1000 ng/µL in DCM) to a paper strip (4mm by 15mm) and allowing the solvent to evaporate for 15 s before placing the strip into a clean odour-delivery pipette (2 mL, 4 mm inner diameter). For each preparation, we severed one antenna of a male, cut the tip open with iris scissors, and mounted it between the glass reference electrode and the recording electrode, both containing insect Ringer’s solution. The odour was puffed at the antenna for 3 s with a custom, flow-rectified odour stimulus controller. We waited for 30 s between each stimulus.

In GC-EAD tests, the stimulus was the outflow from the GC column, which was diluted and delivered to the antennal preparation with a clean, wet, and static-free air flow at 37 cm/s to remove any dead volume that would hamper the clear separation of compounds. In the GC-EAD test after GC-FID analysis, the GC column was removed from the FID, and the separated compounds were directly delivered to the EAD with a custom, 40 cm long heated (250 °C) transfer line. The EAD signal was recorded with HP 34465 A digital multimeter (Keysight, US). Both EAD and FID signal data were aligned, when reconstructing the chromatographs, with Chemstation (Agilent, US) and BenchVue (Keysight, USA) software. GC conditions were identical to those used for quantification, except that the oven ramp was set as 80 °C for 2 min, then 10 °C/min to 230 °C for 10 min. We used dichloromethane (DCM) as a solvent for our test compounds and also tested antennal responses to DCM alone.

### Pheromone quantification

Since both sex pheromone components are carbonic acids (see Results), we used 0.5 mL 0.5 mol/L NaOH solution to thoroughly wash the whole abdomen cuticle of a mature virgin gyne by immersing its abdomen only in a glass tube shaken with a Vortex 3000 (Wiggens, German) for 15 s. The solution was acidified with drops of 1 mol/L HCl to pH 3.0 (pH shown by a 1 μL phenolphthalein/methanol indicator dye). The mixture was then cooled to 5 °C, extracted twice with 20 μL diethyl ether, and vortexed on ice. The organic layers from multiple extractions were combined, dried over anhydrous magnesium sulphate, and concentrated to 10 μL. We took 1 μL of this concentrate for GC analysis.

To determine the efficiency of sex pheromone recovery, we applied 10 μg of synthetic chemical standards (about one gyne equivalent of synthetic sex pheromones) to the inter-segmental cuticle of a model, a freshly frozen male (since males do not produce male-attracting sex pheromone), and extracted it as described above. We then created GC-FID external standard curves. We used the calculated recovery efficiency and these standard curves to quantify the two components.

To determine if the sex pheromone is retained by gynes after mating (and could therefore potentially serve as a queen pheromone), we overwintered six naturally mated gynes from December to April of the subsequent year, when they became foundresses. We used the SPME swipe method to analyse the contents of the sixth intersegmental sternal gland.

### Statistical analyses

All statistical analyses were conducted with SPSS software (IBM, US). We used GLM with a Poisson log-link distribution to test the effect of different putative sex pheromone extracts on the number of attracted males. We made pairwise comparisons of means with Wald chi-square tests and, where appropriate, corrected our significance levels with the Sequential Bonferroni^SB^ method to avoid Type I statistical error.

EAG responses were logistically transformed (TREAG) to fit the linear model. The effects of dose and compound type on hornets were analysed using two-way ANOVA. The TREAG responses were analysed using one-way Repeated-Measures ANOVA to determine the dose effect of each compound because each antenna was repeatedly tested with ascending levels of the same compound. We used Dunnett’s tests to determine which doses elicited responses that were significantly higher than the response to the dichloromethane (DCM) control solvent.

We used ANOVA to analyse the effect of time upon the quantities and ratios of pheromone compounds produced by gynes. To determine the compound ratios that would attract the most males, we log-transformed the number of males attracted and ran an ANOVA with treatment as a fixed effect and research site as a random effect. Per model, we made all pairwise comparisons with Tukey’s Honestly Significant Difference (HSD) tests, which are corrected for Type I error. We report means ± 1 standard error.

### Data accessibility

All data are provided in the Supplementary Materials.

## Results

### General observations on mating behaviour

From September to December, males and gynes emerged from colonies to mate at our field sites. Mature virgin gynes left their nests on sunny days and performed male-calling behaviours in tree canopies in open areas, Male Congregation Area (MCA) sites. Calling gynes rubbed both their sternal and tergal abdomens with their metathoracic legs, thereby appearing to spread sex pheromone over their abdomens. Our observations of males suggested that a slight breeze (0.3 to 3.0 m/s) may enhance the attractiveness of a calling gyne by increasing pheromone dispersion. We observed multiple males swarming over the line of trees in search of gyne sex pheromone. Once a male successfully found a gyne and was accepted, copulation occurred.

**Figure 1:**
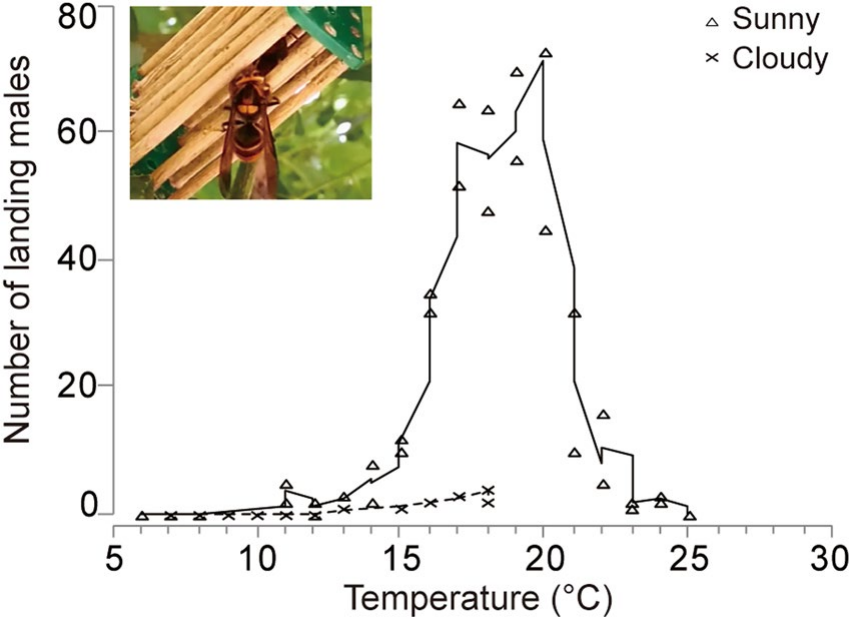
The role of weather (light levels and ambient air temperature) on *Vespa velutina* male attraction to living, mature virgin queens (gynes). The inset photo shows a male landing on a cage with a living gyne. The number of males at a MCA was strongly influenced by weather, primarily by average air temperature during mating attraction and sunlight (sunny = 99.0 ± 4.2 klx, cloudy = 28.8 ± 6.26 klx). The curve fit is a moving average between two successive points. Sample sizes are given in Table S1.

All of the tested gynes attracted males. The number of *V*. *velutina* males attracted to a gyne significantly increased with air temperature (GLM: *G* ^2^_13_ = 307.66, *P* < 0.001) and light levels (*G* ^2^_1_ = 43.72, *P* < 0.001, cloudy vs. sunny day comparison in Fig. 1) under our study conditions. We found no significant interaction of temperature*light (*G* ^2^_4_ = 2.49, *P* = 0.647 > 0.05). Control cages did not attract any males in any trial (*N* = 50 trials). Good temperature and sunlight conditions for mating at our field sites ranged from 15 °C to 22 °C on sunny days (50–100 klx), typically from 10:00 to 15:00 during the mating season, September to December. Once initiated, male swarming usually persisted for two hours. At higher temperatures, male swarming began earlier in the day but tended to last for less than 2 h.

### Gynes produce sex pheromone in their sixth intersegmental sternal glands

**Figure 2:**
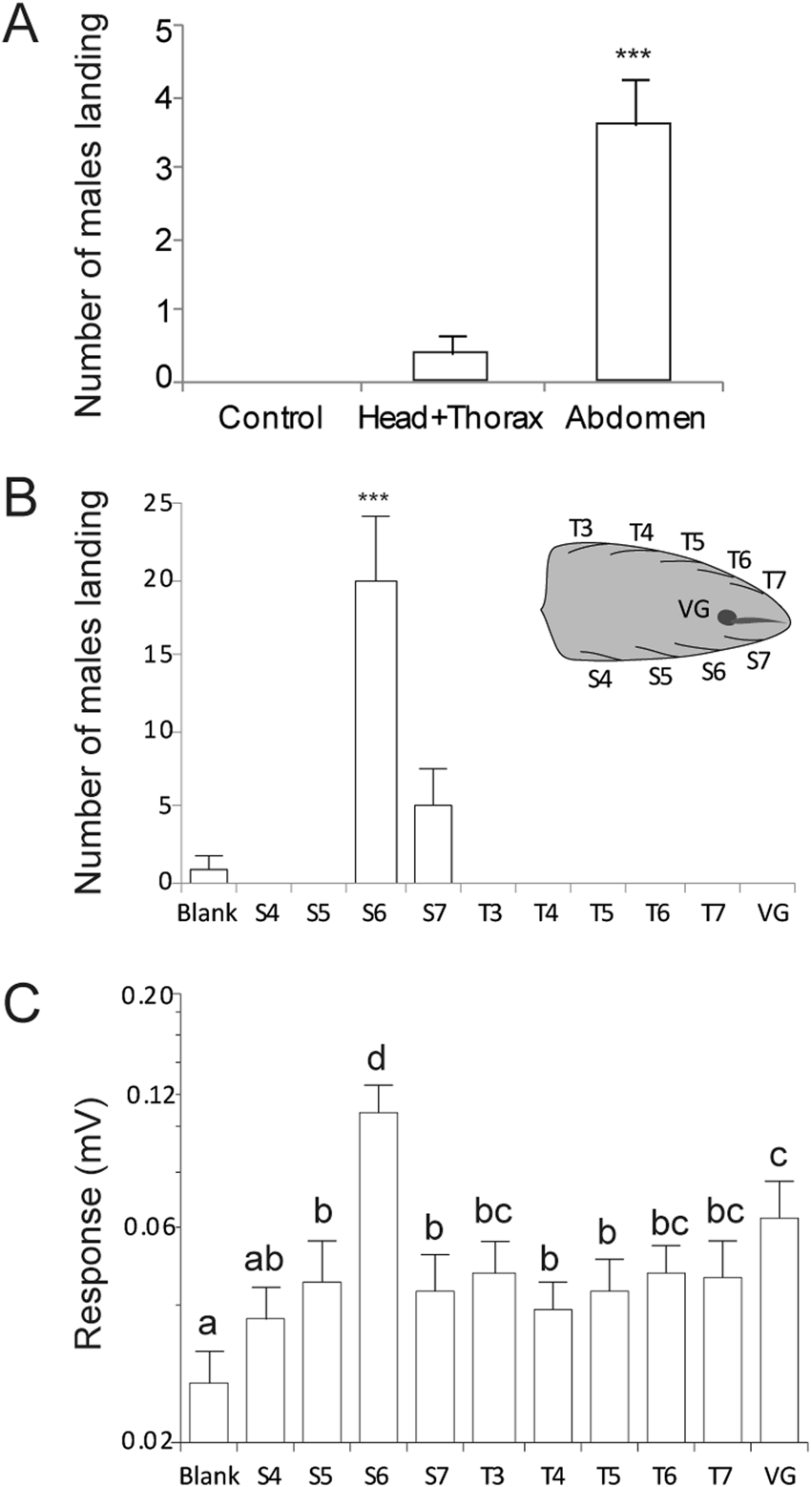
*Vespa velutina* gyne sex pheromone is produced in glands within the sixth intersegmental sternal cuticle. Intersegmental sternal (S) and tergal (T) glands were rubbed with thin filter paper strips to obtain the extracts. VG indicates venom gland extract. Means and standard errors are shown. (**A**) The number of males landing in responses to extracts from major body parts. Only the abdomen was significantly attractive. (**B**) The number of males landing in response to extracts from abdominal glands at different locations (venom gland = VG). Region S6 elicited significantly higher male attraction than extracts from all other locations (***P < 0.001, Sequential Bonferroni corrected). (**C**) Electroantennogram (EAG) responses of males to these extracts. Different letters indicate significant differences (Tukey’s HSD: *P* < 0.05). Sample sizes are given in Table S1.

Our body part assay revealed that males were only strongly attracted to gyne abdomens (body part effect: *G* ^2^_1_ = 15.193, *P* < 0.001, Fig. 2A). There was no influence of field site (*G* ^2^_2_ = 0.610, *P* = 0.737) and the interaction of site*body part (*G* ^2^_2_ = 0.280, *P* = 0.869) was not significant.

We therefore focused on only the abdomen, using filter paper extracts of the intersegmental tergal and sternal cuticles. There was an overall effect of extract location on male attraction (*G* ^2^_3_ = 192.33, *P* < 0.001). Specifically, extract from the sixth intersegmental sternal cuticle glands^40^ attracted significantly more males than extracts from any other location (Wald chi-square test: *P* < 0.0001, Fig. 2B). Male hornet antennal responses (EAG) likewise showed a significant effect of extract location (ANOVA: *F*_*(1*, *8)*_ = 79.96, *P* < 0.001). Again, the extract from the sixth intersegmental sternal cuticle glands of gynes elicited the strongest antennal response (Dunnett’s test: *P* < 0.001, Fig. 2C).

Because males in other species may be attracted to female venom gland compounds, we also tested *V*. *velutina* venom gland extract. Males showed elevated EAG responses to female venom gland extract as compared to the blank control (Fig. 2C) but were not behaviourally attracted to this extract (Fig. 2A). Hornets have multiple olfactory glomeruli^41^ that likely respond to a wide range of compounds, including venom ketone volatiles^35^.

### The major sex pheromone compounds are 4-OOA and 4-ODA

**Figure 3:**
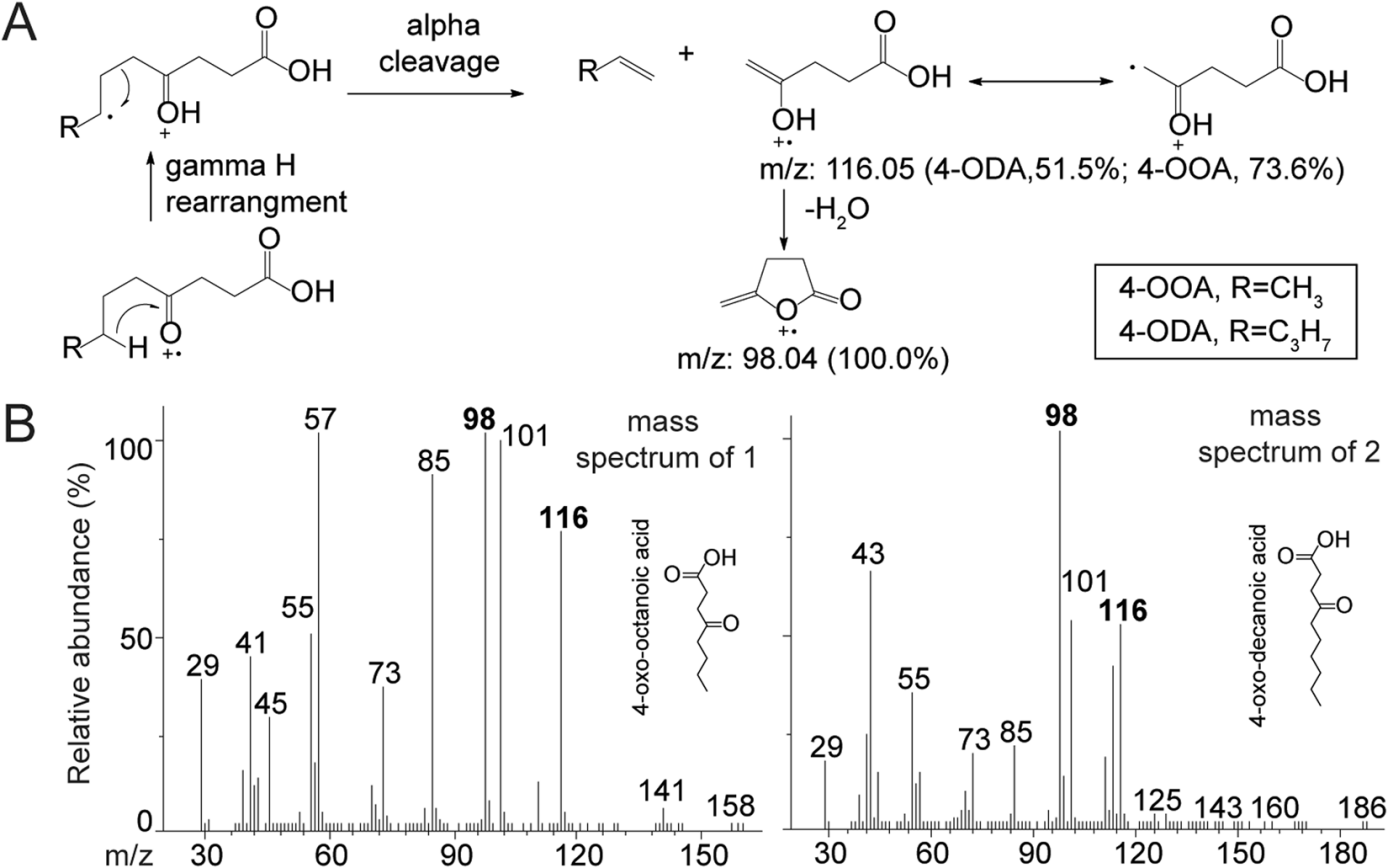
Mass spectra analysis of the two pheromone components. (**A**) Production pathways of the two even-electron ions (m/z 98 and m/z 116) in the mass spectra of the two components. (**B**) The mass spectra of these two components (m/z 98 and m/z 116) in natural sex pheromone are identical to that of pure, synthetic 4-oxo-octanoic acid (4-OOA) and 4-oxo-decanoic acid (4-ODA), respectively. Sample sizes are given in Table S1.

We therefore focused on the compounds produced by the sixth intersegmental sternal cuticle glands. We found two major compounds (peaks 1 and 2) in the cuticular extract from this region. The peaks of the two compounds on a non-polar column had a tailing shape, indicating highly polar components. We therefore used selective acid extraction to determine if they were acids. Based upon the mass spectra, we noticed two even electron ions, *m/z* 98 and *m/z* 116, which would be the product of γ-H rearrangements if the structures were ketoacids. After NaBH_4_ reduction and lactonization, the two compounds were converted to their corresponding lactones (Fig. S3), which provided further confirmation that they were ketoacids. More specifically, the base peak of *m/z* 85 indicated gamma lactones that are derivatives of 4-oxo acids. In addition, the molecular ion peaks of *m/z* 158 and *m/z* 182 respectively suggested that these two compounds were 4-oxo-octanoic acid and 4-oxo-decanoic acid (Fig. 3). We then synthesized pure 4-oxo-octanoic acid and 4-oxo-decanoic acid standards (see Methods), which allowed us to confirm our putative identifications based upon identical retention times and mass spectra.

### Quantification of 4-OOA and 4-ODA in mature virgin gynes

A mature virgin gyne produced an average of 10.38 ± 1.48 μg of 4-OOA and 13.32 ± 1.51 μg of 4-ODA at a fairly constant ratio of 0.78 ± 0.02. Our temporal analyses showed that 4-OOA and 4-ODA were also produced in the sixth intersegmental cuticle glands at approximately the same level throughout the day (ANOVA: *F*_(9,24)_ = 1.823, *P* = 0.12). Moreover, the ratio of these two components was consistent and did not significantly vary with time of day (*F*_(9, 24)_ = 0.994, *P* = 0.47, Fig. S4). However, the production of both components ceased by the end of the mating season. No 4-OOA or 4-ODA was detected after the mated gynes became foundresses (Fig. S4).

### 4-OOA and 4-ODA elicited strong antennal responses in males and attracted males in bioassays

**Figure 4:**
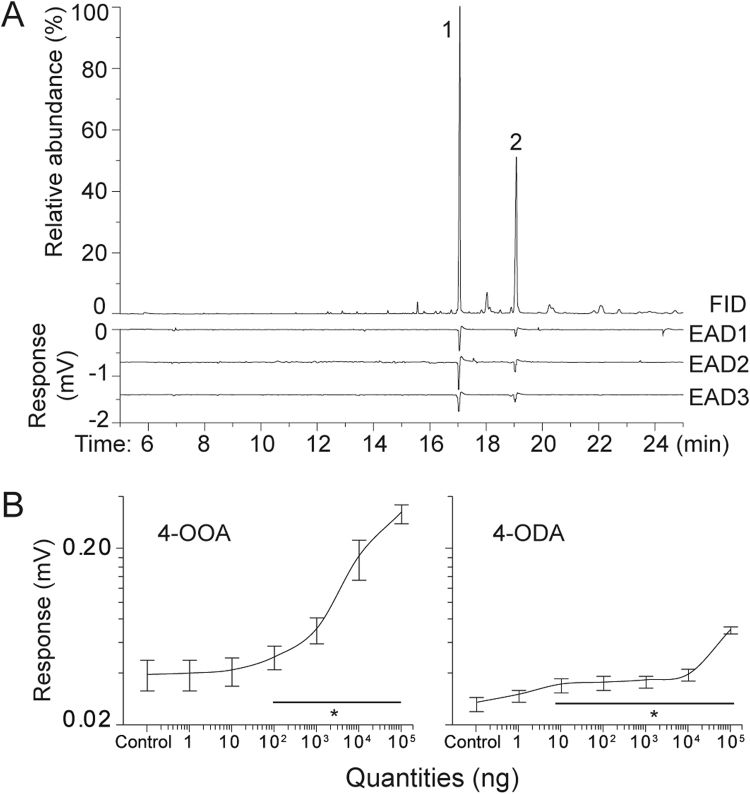
*Vespa velutina* gyne sex pheromone consists of 4-OOA and 4-ODA, as determined by Solid Phase Microextraction (SPME) and male antennal responses (EAG). (**A**) SPME of the sternal sixth intersegmental cuticle revealed two peaks (1 and 2) and corresponded to two highly consistent male antennal responses (EAD, signal/noise ratio > 3, 9 replicates). We show three representative antennal signals from three different males. (**B**) Male antennal responses to different doses of each key compound. Quantities that elicited a significantly higher response than the control are shown by a line and marked with an asterisk (*P < 0.05). Means and standard errors are shown. Sample sizes are given in Table S1.

We next examined the dose-dependent antennal responses of males to the pure chemical standards that we synthesized. 4-OOA and 4-ODA generated highly reproducible electrophysiological responses in our GC-EAD tests of males (Fig. 3A). As expected, both compounds elicited stronger antennal responses at higher quantities: 4-OOA (ANOVA: *F*_(1, 11)_ = 237.62, *P* < 0.001) and 4-ODA (*F*_(1, 11)_ = 540.84, *P* < 0.001, Fig. 4B). The minimum quantities that elicited a significantly greater response, as compared to the control, were 100 ng for 4-OOA (*P* = 0.038) and 10 ng for 4-ODA (*P* = 0.012). Thus, the quantities of 4-OOA and 4-ODA produced by mature virgin gynes are 100- to 1000-fold greater than the minimum detection differences exhibited by male antennae.

### Significantly more males were only attracted to the natural gyne ratio of 4-OOA and 4-ODA

**Figure 5:**
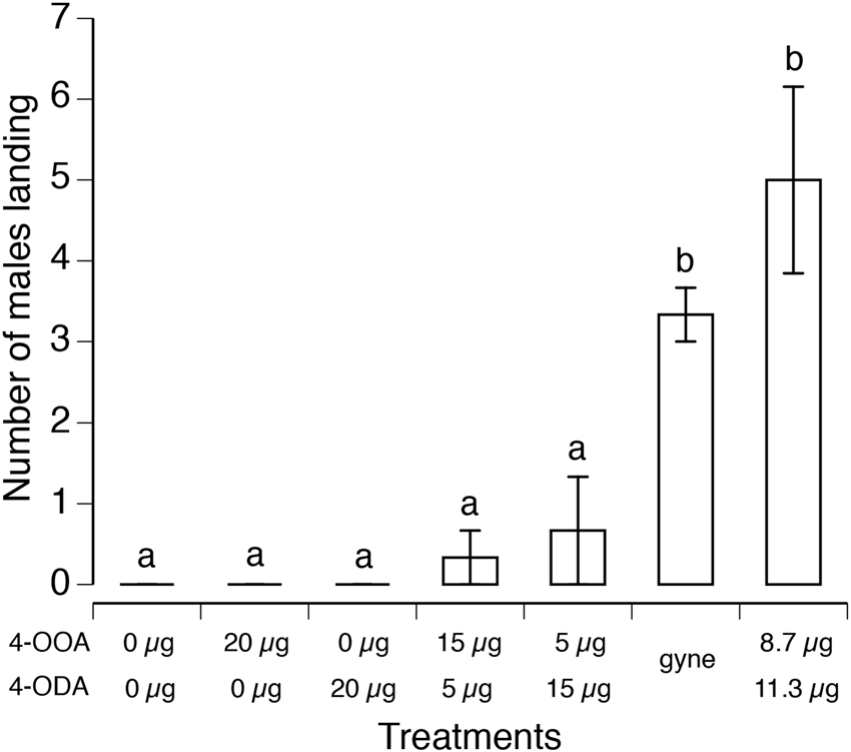
For males, the most attractive ratio of sex pheromone compounds is a natural ratio. A 0.77 ratio of 4-OOA/4-ODA (very similar to the mean 0.78 ratio found in gynes) was highly attractive to males and did not attract a significantly different number of males as compared to a live, mature female gyne. No other ratios attracted significantly more males than the blank control. Different letters indicate significant differences, Tukey HSD test (*P* < 0.05). Means and standard errors are shown. Sample sizes are given in Table S1.

We then tested the effects of different ratios of 4-OOA and 4-ODA on male attraction (Fig. 5). There was a significant effect of treatment (ANOVA: *F*_(6,12)_ = 16.73, *P* < 0.0001, field site accounted for <1% of model variance). For males, a natural compound ratio found in gynes (a 0.77 ratio of 4-OOA/4-ODA = 8.7 µg/11.3 µg) was the only ratio that attracted significantly more males than the control and was not significantly different from attraction to a live female gyne. None of the other ratios attracted significantly more males than the blank control (Tukey HSD test, *P* < 0.05).

## Discussion

Pheromones are chemical signals that have evolved to convey information between different individuals of the same species^21^. Sex pheromones play a key role in the mating and therefore fitness of multiple animal species. We demonstrate that *V*. *velutina* gynes produce two compounds, 4-oxo-octanoic acid (4-OOA) and 4-oxo-decanoic acid (4-ODA), in a ratio of 0.78 from their sixth intersegmental sternal glands. Male antennae were highly sensitive to these compounds (detecting 1/100 to 1/1000 of the amounts produced by a gyne), and males were highly and significantly attracted to pure synthetic compounds at the natural ratio, but not at different ratios. These are all classic characteristics of an insect sex pheromone^21^. After gynes mated, they no longer produced these compounds, demonstrating that this chemical signal is also time-limited to mating. If these compounds were simply olfactory cues and not information that has evolved to allow males to find females, they should not elicit such strong and consistent male responses that depend upon a precise ratio and they would unlikely be produced only when females need to mate.

These results also provide the first demonstration of an effective way to lure *V*. *velutina* males. This synthetic sex pheromone should be useful for helping to control this invasive species and, particularly, in monitoring new infestations and delineating invasion fronts. The design and testing of effective sex pheromone traps can now proceed based upon these identified compounds. A standard, triangular glue trap^9^ (an open triangular prism consisting of three paper walls with glue and the pheromone lure on one wall) may be effective. During the mating season, males would be drawn in and then caught by touching the glue, a non-toxic compound. Males do not produce alarm pheromones^35,39^ and caught males should therefore not be able to warn about the trap. To lure males, a visual dummy may be unnecessary. In preliminary trials, we found that males would approach a filter paper bait consisting of sternal gland secretions alone. In our experiment, we opted to measure landings as a more rigorous way of quantifying male choice. However, males seeking gynes often needed to enter the enclosing shelter of leaves around the gyne. This behaviour suggests that an enclosed trap providing only sex pheromone could work. If visual dummies are necessary, they may be fabricated. We note that the first male to enter the trap becomes, in essence, a model to attract other males (Fig. 2A,B). We will test different trap designs in a future study.

In the termites (Isoptera), some species evidently produce sex pheromone in their sternal glands^23,24^. However, to our knowledge, prior studies have not shown that any Hymenoptera can produce sex pheromones in their sternal glands, although Hymenoptera are known to produce sex pheromones in their mandibular^42–44^ and venom^11,12,16,28^ glands. Our study therefore provides the first evidence that the sternal glands^45^ of Hymenoptera can secrete a long-distance sex pheromone.

As in the mating systems of many animals, multimodal information from multiple sources is likely important^46^. Our preliminary results suggested that filter paper with sex pheromone and a visual model are both necessary for males to land. Once a male is close to a gyne, visual recognition evidently plays a role. Additional sources of odour information may also be involved, providing odour recognition or even a short-distance sex pheromone. There are slightly differences between the ratios of some minor cuticular hydrocarbons, particularly the more volatile ones, between males and gynes (Fig. S1). Other short-range odours, such as compounds produced by antennal glands^18^, may facilitate gyne recognition.

Recently, Couto *et al*.^41^ demonstrated that the overall organization of the *V*. *velutina* brain is quite similar in queens, workers, and males. In females, the antennal lobe, which processes olfactory information, has about 256 glomeruli in nine clusters. However, the antennal lobe of males shows some marked differences because it contains fewer glomeruli with a different clustering^41^. Such differences between males and females are common in social Hymenoptera^41^. Given our results, it would be interesting to measure *V*. *velutina* antennal lobe responses to 4-OOA and 4-ODA, individually and at different ratios. Single sensillum recording of the antennae alone would also be enlightening.

In Hymenoptera, sex compounds have evolved from a diverse set of compounds and sources. Both 4-OOA and 4-ODA are oxo acids and have been previously identified in leaf-cutting ants as worker secretions with an unknown function, in the metapleural gland secretions of neotropical leaf cutting ants^38^ (where they act as antimicrobials^47^ but not sex pheromones), in honey bees as sex and queen pheromones^42–44^, in Scoliid wasps as sex pheromones^22,48^, and in some African Danainae butterflies as male scent organ odours (potential sex-related pheromones)^49^. It seems unlikely that 4-OOA and 4-ODA share a common evolutionary origin in groups as diverse as Apini, Scolidae, Danainae, and *Vespa*. However, these two compounds may be used as sex pheromones by related *Vespa* species. To facilitate such investigations, we note that these highly polar compounds cannot be extracted by non-polar solvents^13,50^. We recommend SPME and suggest selective extraction of specific body parts with clean filter paper strips followed by NaOH extraction of these paper strips to ensure a clear separation of acids and the abundant cuticular hydrocarbons that will be removed by rubbing. With this data, it may be possible to form a clearer evolutionary picture of *Vespa* sex pheromones.

In honey bees, sex pheromones can also serve as queen pheromones, although the cocktail of different queen pheromone compounds (proportions and different chemicals) are known to dramatically change as a result of queen mating, thereby providing an honest signal of queen quality to workers^51,52^. Given this, it may not be surprising that *V*. *velutina* female sex pheromone compounds did not persist in mated queens, foundresses (Fig. S4). We suspect that mated *V*. *velutina* foundresses produce a different set of queen pheromones, a topic deserving of further study.

## Electronic supplementary material

Supplemental Information
