# Supplementary material for: The sex pheromone of a globally invasive honey bee predator, the Asian eusocial hornet, Vespa velutina
Source: Sci Rep. 2017 Oct 11;7:12956. doi: 10.1038/s41598-017-13509-7 (PMC5636794; doi:10.1038/s41598-017-13509-7)
Supplement: Supplementary file 1 — Supplemental Information [file 41598_2017_13509_MOESM1_ESM.pdf]

Supplemental Information

The Sex pheromone of a globally invasive honey bee predator, the Asian eusocial hornet, *Vespa velutina*

Ping Wen[1*#], Ya-Nan Cheng[1,2#], Shi-Hao Dong[3], Zheng-Wei Wang[1], Ken Tan[1*], James C. Nieh[4]

[#]These authours contribute equally to this work.

*Corresponding authours: kentan@xtbg.ac.cn, wenping@xtbg.ac.cn

[1]Key Laboratory of Tropical Forest Ecology, Xishuangbanna Tropical Botanical Garden, Chinese Academy of Sciences, Kunming, Yunnan Province, 650223, China

[2]University of Chinese Academy of Sciences, Beijing, 100049, China

[3]Eastern Bee Research Institute, Yunnan Agricultural University, 650201 Kunming, China

[4]Division of Biological Sciences, Section of Ecology, Behaviour, and Evolution, University of California, San Diego, La Jolla, California, USA

Table S1. Sample sizes for all experiments. In total, we used 10 different colonies over one year in Kunming at Yunnan Agriculture University (YAU) and the Southwest Biodiversity Research Center (SBRC). A Male Congregation Area is abbreviated as "MCA".

Experiment	Sites	Year	No. of colonies/MCA	No. of hornets used per trial	No. of replicates per colony/MCA/hour	Total no. of individual hornets
**Observation trapping**	YAU, SBRC	2016	3 colonies for gynes >6 colonies* providing males from two MCAs	12 gynes in each MCA.	3 in each MCA	36 gynes and 668 attracted males
**Bioassay of body parts**	YAU, SBRC	2016	3 colonies for gynes >6 colonies providing males from two MCAs	1 gyne for dissection, 1 gyne for extraction and 1 male for the model	3	18 gynes extracted, 9 male models 63 males attracted
**EAG tests of body part extracts**	YAU, SBRC	2016	3 colonies for gynes >6 colonies* providing males from two MCAs	1 gyne for extraction and 1 male for detection	3	9 gynes and 9 males
**SPME-GC of pheromone production**	YAU, SBRC	2016	3 colonies	1 gyne	3 to 5 analyses per hour	33 gynes
**GC-MS of gyne gland**	YAU, SBRC	2016	3 colonies	2 gynes	3	18 gynes
**GC-EAD of gyne sternal gland**	YAU, SBRC	2015	3 colonies for gynes >6 colonies* for males from two MCAs	1 gyne for extraction and 1 male for detection	3	9 gynes and 9 males
**EAG tests of STDs**	YAU, SBRC	2016	>6 colonies from two MCA	1 male per component (two components tested)	4	24 males
**Bioassay of synthetic chemical standards**	YAU, SBRC	2016	3 colonies for gynes >6 colonies* for males from two MCAs	1 living gyne control, 1 male as a model	4 and 5 in two MCA	9 gynes used for control. 9 male models. 31 males attracted
**Microscale chemistry of extracts**	YAU, SBRC	2016	Gynes from 3 colonies	1	3	9 gynes
**SPME-GC of pheromone production**	YAU, SBRC	2017	3 incipient colonies	1 foundress	2	6 foundresses

*Estimated based upon the known number of colonies in the local area. Hornet colonies are large and relatively conspicuous at YAU and SBRC.

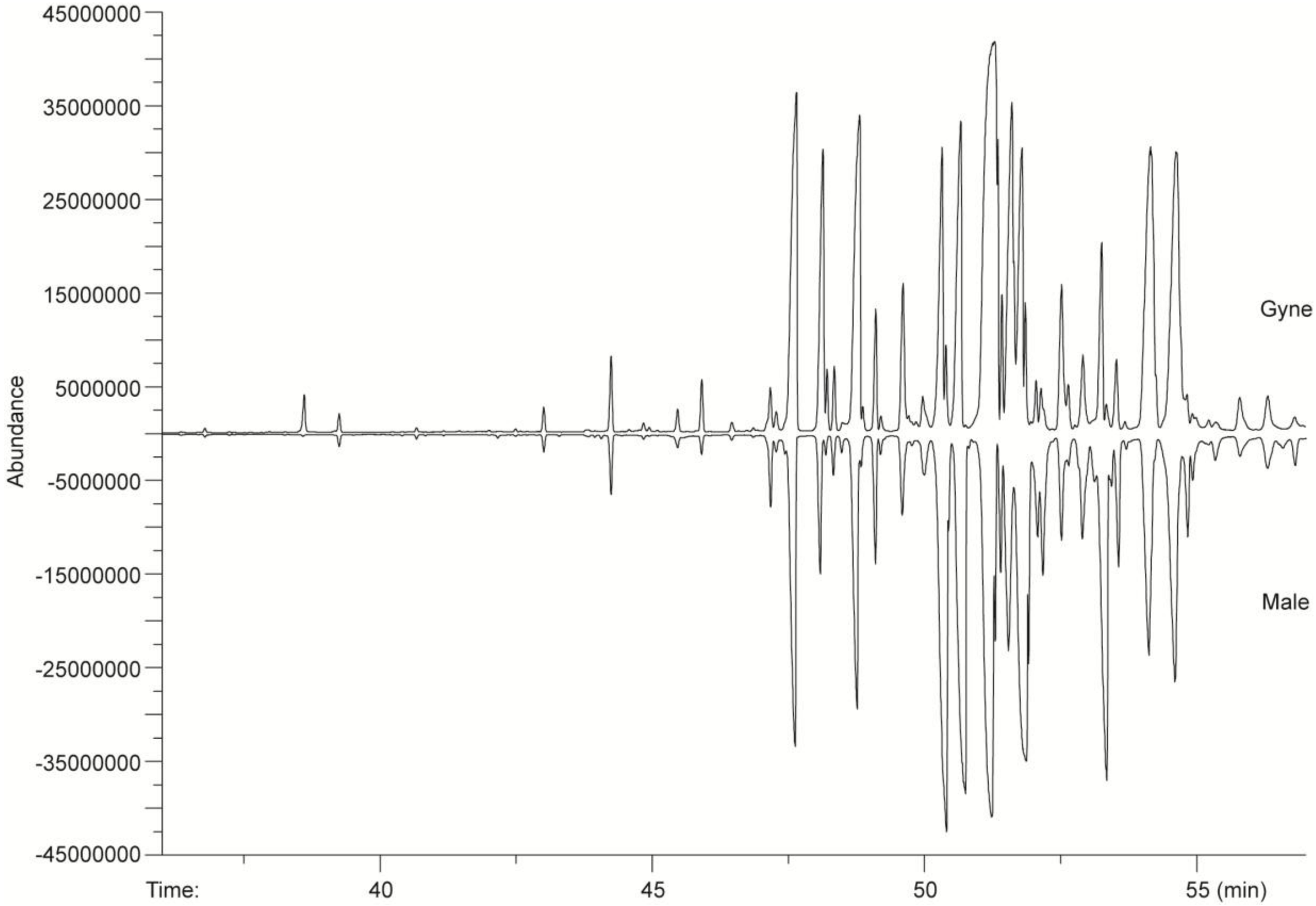

Figure S1. Males are suitable as an olfactory model for a female gyne because both share similar (non-sex pheromone) cuticular hydrocarbons (CHC). Comparison of the CHC between the male and gyne of *Vespa velutina*. Total Ion Current Chromatograms (TICs) were obtained from whole body n-hexane washes. Similar CHC profiles were observed in both sexes.

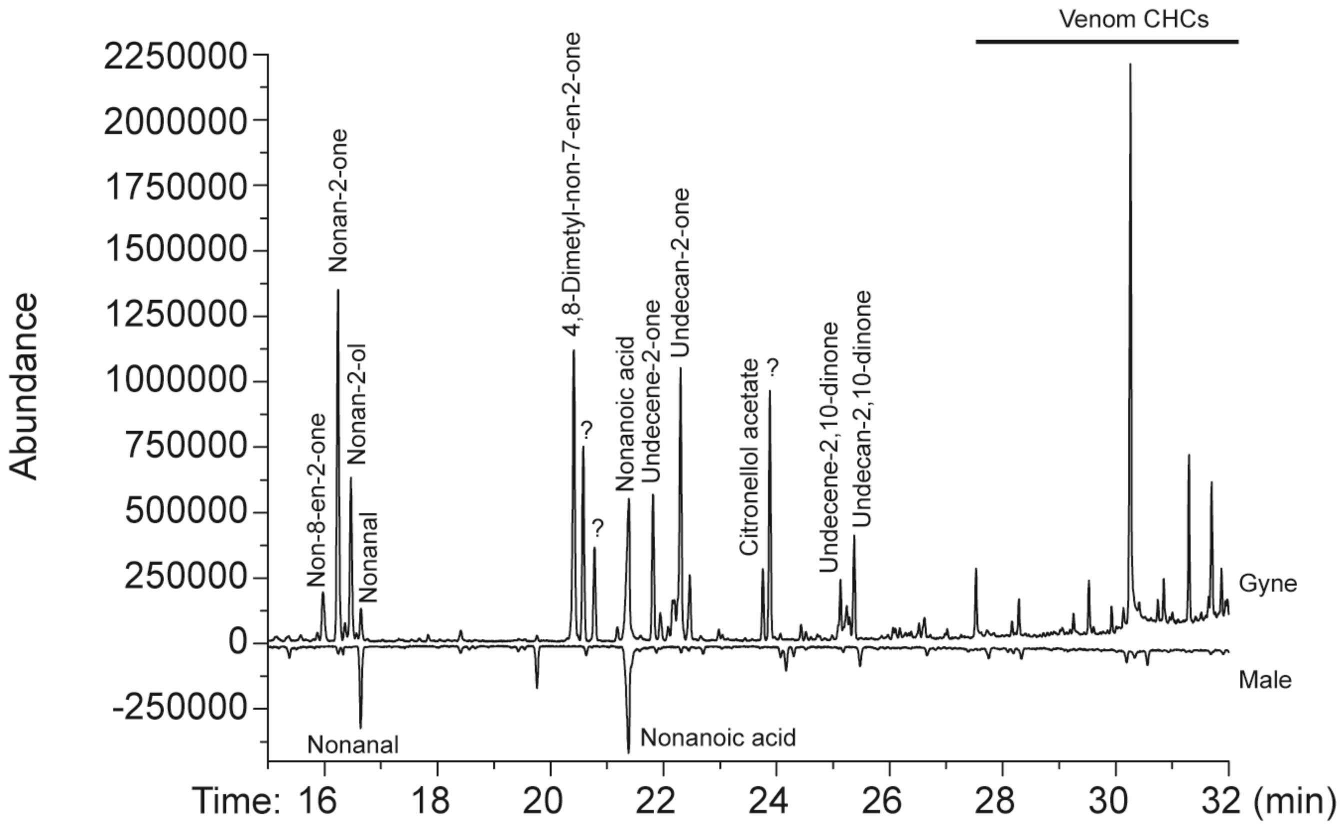

Figure S2. Males are also suitable as an olfactory model of a female gyne because males do not produce any alarm pheromone. Comparison of the TICs of n-hexane extracts of gyne sting venom (alarm pheromone) extract (the upper trace) and male whole-body n-hexane extract (the lower trace). Males produce nonanal and nonanoic acid, but these are not reported to be alarm compounds.

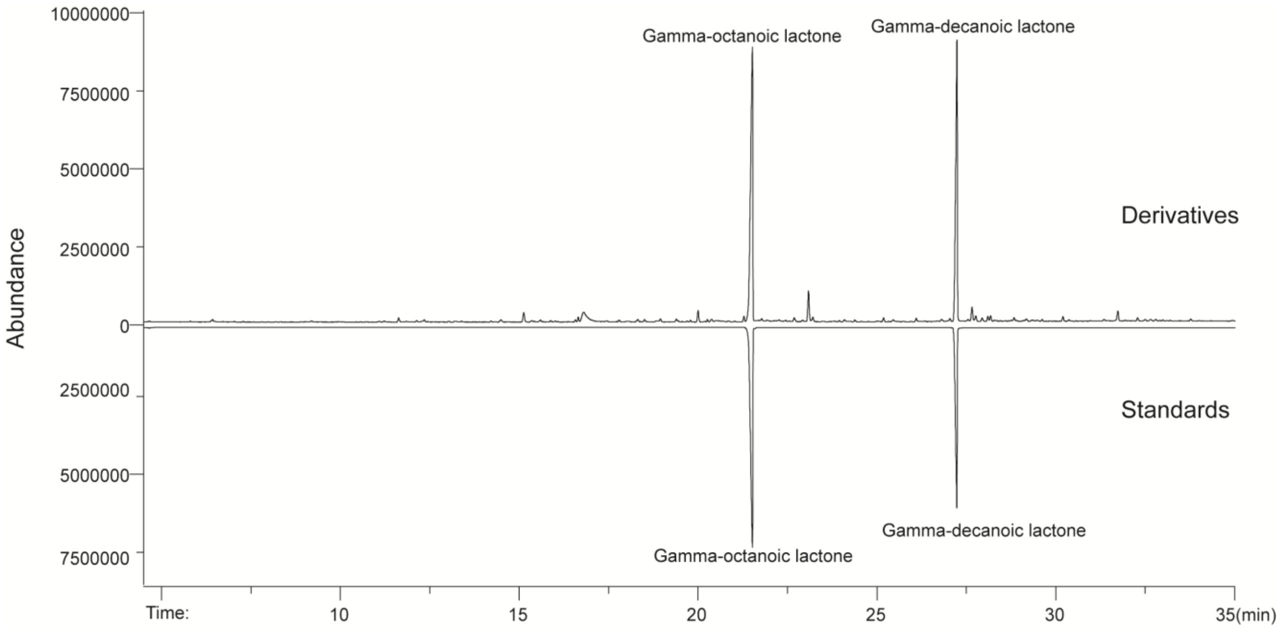

Figure S3. Identification of sex pheromone compounds. GC-MS analysis of HS-SPME extracts of the microchemical lactonized derivatives of extracted sex pheromone (upper trace). The two oxo acids shown were converted into corresponding lactones and identified based upon comparison with the retention times and mass spectra of pure synthetic chemical standards (lower trace).

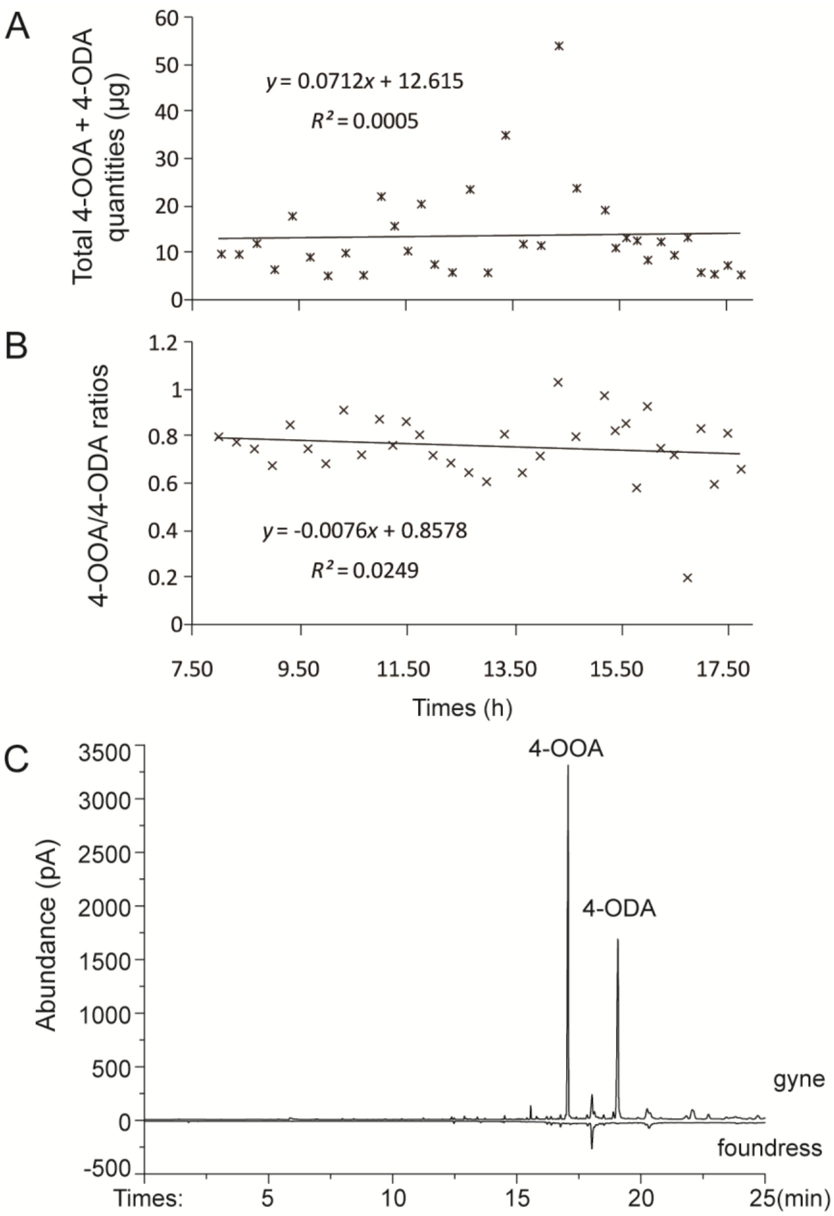

Figure S4. Temporal dynamics of pheromone production. (A) The quantities of 4-OOA and 4-ODA from the gland were not correlated with time of day. (B) In addition, the ratios of two components were not correlated with the time of day. Linear regression lines and equations are shown. C. Sex pheromone production ceases once a gyne becomes a foundress. Comparative GC analysis of SPME wiping extracts from the sixth intersegmental sternal glands of a gyne and a foundress. Representative chromatograms shown.

CRUDE DATA FOR FIGURES

Figure 1

Observes	Temperature	Illuminance	Light intensity (klx)	Number of landing males	Control
36	7	Cloudy	9.3	0	0
39	7	Cloudy	8.5	0	0
37	8	Cloudy	6.7	0	0
38	9	Cloudy	7.9	0	0
40	10	Cloudy	10	0	0
44	10	Cloudy	13	0	0
43	11	Cloudy	12	0	0
41	12	Cloudy	17	0	0
45	12	Cloudy	11	0	0
42	13	Cloudy	43	1	0
46	15	Cloudy	71	1	0
47	16	Cloudy	56	2	0
48	17	Cloudy	67	3	0
49	18	Cloudy	43	4	0
50	18	Cloudy	57	2	0
6	6	Sunny	46	0	0
21	6	Sunny	83	0	0
5	7	Sunny	52	0	0
20	7	Sunny	93	0	0
1	8	Sunny	65	0	0
22	8	Sunny	87	0	0
3	11	Sunny	93	2	0
35	11	Sunny	75	5	0
2	12	Sunny	67	0	0
19	12	Sunny	98	2	0
4	13	Sunny	73	3	0
8	14	Sunny	63	8	0
28	14	Sunny	98	2	0
7	15	Sunny	76	12	0
18	15	Sunny	98	10	0
17	16	Sunny	103	32	0
24	16	Sunny	105	35	0
9	17	Sunny	91	52	0
25	17	Sunny	87	65	0
10	18	Sunny	112	48	0
31	18	Sunny	84	64	0
12	19	Sunny	95	56	0
23	19	Sunny	125	70	0
26	20	Sunny	105	73	0
32	20	Sunny	112	45	0
11	21	Sunny	125	32	0
30	21	Sunny	129	10	0
33	22	Sunny	121	5	0
34	22	Sunny	118	16	0
13	23	Sunny	135	2	0
27	23	Sunny	131	1	0

14	24	Sunny	138	3	0
15	24	Sunny	129	2	0
16	25	Sunny	125	0	0
29	25	Sunny	127	0	0

Figure 2A Dissection bioassay

Sites	Gynes	Bodypart	Nofmales
1	1	Control	0
1	1	H+T	1
1	1	Abdomen	3
1	2	Control	0
1	2	H+T	0
1	2	Abdomen	4
1	3	Control	0
1	3	H+T	0
1	3	Abdomen	3
2	4	Control	0
2	4	H+T	1
2	4	Abdomen	5
2	5	Control	0
2	5	H+T	0
2	5	Abdomen	4
2	6	Control	0
2	6	H+T	0
2	6	Abdomen	2
3	7	Control	0
3	7	H+T	1
3	7	Abdomen	5
3	8	Control	0
3	8	H+T	1
3	8	Abdomen	4
3	9	Control	0
3	9	H+T	0
3	9	Abdomen	3

Figure 2B Gland filter paper extract bioassay

Samples	Sites	Nof males
control	1	1
S4	1	0
S5	1	0
S6	1	7
S7	1	2
T3	1	0
T4	1	0
T5	1	0
T6	1	0
T7	1	0
Sting	1	0
control	2	0
S4	2	0
S5	2	0
S6	2	5
S7	2	1
T3	2	0
T4	2	0
T5	2	0
T6	2	0
T7	2	0
Sting	2	0
control	3	0
S4	3	0
S5	3	0
S6	3	8
S7	3	2
T3	3	0
T4	3	0
T5	3	0
T6	3	0
T7	3	0
Sting	3	0

Figure 2C Gland EAG bioassay

No.	SH name	Tested Part	Response (mV)	LogResponse+2.1
1	1	control	0.0162	0.3093
2	1	S4	0.0191	0.3798
3	1	S5	0.0181	0.3576
4	1	S6	0.0857	1.0331
5	1	S7	0.0191	0.3798
6	1	T3	0.0252	0.5021
7	1	T4	0.0381	0.6809
8	1	T5	0.0238	0.4768
9	1	T6	0.0286	0.5559
10	1	T7	0.0191	0.3798
11	1	ST	0.0357	0.6528
12	2	control	0.0095	0.0788
13	2	S4	0.0110	0.1395
14	2	S5	0.0143	0.2549
15	2	S6	0.0476	0.7778
16	2	S7	0.0167	0.3219
17	2	T3	0.0191	0.3798
18	2	T4	0.0143	0.2549
19	2	T5	0.0124	0.1928
20	2	T6	0.0214	0.4310
21	2	T7	0.0152	0.2829
22	2	ST	0.0224	0.4499
23	3	control	0.0167	0.3219
24	3	S4	0.0191	0.3798
25	3	S5	0.0129	0.2091
26	3	S6	0.1048	1.1202
27	3	S7	0.0286	0.5559
28	3	T3	0.0286	0.5559
29	3	T4	0.0200	0.4010
30	3	T5	0.0262	0.5181
31	3	T6	0.0238	0.4768
32	3	T7	0.0286	0.5559
33	3	ST	0.0381	0.6809
34	4	control	0.0262	0.5181
35	4	S4	0.0333	0.6229
36	4	S5	0.0333	0.6229
37	4	S6	0.0571	0.8570
38	4	S7	0.0286	0.5559
39	4	T3	0.0395	0.6969
40	4	T4	0.0333	0.6229
41	4	T5	0.0295	0.5702
42	4	T6	0.0381	0.6809
43	4	T7	0.0381	0.6809
44	4	ST	0.0314	0.5973
45	5	control	0.0238	0.4768
46	5	S4	0.0333	0.6229
47	5	S5	0.0500	0.7990
48	5	S6	0.1095	1.1395
49	5	S7	0.0333	0.6229
50	5	T3	0.0429	0.7320
51	5	T4	0.0262	0.5181
52	5	T5	0.0333	0.6229
53	5	T6	0.0381	0.6809
54	5	T7	0.0381	0.6809

55	5	ST	0.0524	0.8192
56	6	control	0.0262	0.5181
57	6	S4	0.0429	0.7320
58	6	S5	0.0571	0.8570
59	6	S6	0.1095	1.1395
60	6	S7	0.0452	0.7555
61	6	T3	0.0452	0.7555
62	6	T4	0.0410	0.7123
63	6	T5	0.0452	0.7555
64	6	T6	0.0524	0.8192
65	6	T7	0.0429	0.7320
66	6	ST	0.0652	0.9145
67	7	control	0.0429	0.7320
68	7	S4	0.0714	0.9539
69	7	S5	0.0952	1.0788
70	7	S6	0.1238	1.1928
71	7	S7	0.0714	0.9539
72	7	T3	0.0857	1.0331
73	7	T4	0.0714	0.9539
74	7	T5	0.0810	1.0082
75	7	T6	0.0857	1.0331
76	7	T7	0.0952	1.0788
77	7	ST	0.1048	1.1202
78	8	control	0.0524	0.8192
79	8	S4	0.0762	0.9819
80	8	S5	0.0952	1.0788
81	8	S6	0.2286	1.4590
82	8	S7	0.0952	1.0788
83	8	T3	0.0857	1.0331
84	8	T4	0.0619	0.8917
85	8	T5	0.0810	1.0082
86	8	T6	0.0810	1.0082
87	8	T7	0.0857	1.0331
88	8	ST	0.1286	1.2091
89	9	control	0.0286	0.5559
90	9	S4	0.0333	0.6229
91	9	S5	0.0333	0.6229
92	9	S6	0.0952	1.0788
93	9	S7	0.0476	0.7778
94	9	T3	0.0524	0.8192
95	9	T4	0.0524	0.8192
96	9	T5	0.0595	0.8747
97	9	T6	0.0595	0.8747
98	9	T7	0.0595	0.8747
99	9	ST	0.0857	1.0331

Figure 4B

Colony	Bee Name	Subject	Quantity/ng	Response (mV)	LogResponse+2.1
1	1	1-1	control	0.0216	0.4350
1	1	1-1	1	0.0286	0.5559
1	1	1-1	10	0.0238	0.4768
1	1	1-1	100	0.0333	0.6229
1	1	1-1	1000	0.1095	1.1395
1	1	1-1	10000	0.1429	1.2549
1	1	1-1	100000	0.4286	1.7320
1	2	1-2	control	0.0333	0.6229
1	2	1-2	1	0.0351	0.6455
1	2	1-2	10	0.0381	0.6809
1	2	1-2	100	0.0381	0.6809
1	2	1-2	1000	0.1048	1.1202
1	2	1-2	10000	0.1286	1.2091
1	2	1-2	100000	0.3905	1.6916
1	3	1-3	control	0.0264	0.5214
1	3	1-3	1	0.0286	0.5559
1	3	1-3	10	0.0238	0.4768
1	3	1-3	100	0.0310	0.5907
1	3	1-3	1000	0.0357	0.6528
1	3	1-3	10000	0.0762	0.9819
1	3	1-3	100000	0.2095	1.4212
1	4	1-4	control	0.0714	0.9539
1	4	1-4	1	0.0667	0.9239
1	4	1-4	10	0.0714	0.9539
1	4	1-4	100	0.0762	0.9819
1	4	1-4	1000	0.1048	1.1202
1	4	1-4	10000	0.5000	1.7990
1	4	1-4	100000	0.5714	1.8570
2	1	2-1	control	0.1048	1.1202
2	1	2-1	1	0.1095	1.1395
2	1	2-1	10	0.1048	1.1202
2	1	2-1	100	0.1190	1.1757
2	1	2-1	1000	0.1619	1.3093
2	1	2-1	10000	0.5476	1.8385
2	1	2-1	100000	0.5714	1.8570
2	2	2-2	control	0.0214	0.4310
2	2	2-2	1	0.0208	0.4188
2	2	2-2	10	0.0214	0.4310
2	2	2-2	100	0.0286	0.5559
2	2	2-2	1000	0.0429	0.7320
2	2	2-2	10000	0.1905	1.3798
2	2	2-2	100000	0.3810	1.6809
2	3	2-3	control	0.0312	0.5935
2	3	2-3	1	0.0304	0.5823
2	3	2-3	10	0.0333	0.6229
2	3	2-3	100	0.0381	0.6809
2	3	2-3	1000	0.0452	0.7555
2	3	2-3	10000	0.0857	1.0331
2	3	2-3	100000	0.1905	1.3798
2	4	2-4	control	0.0264	0.5214
2	4	2-4	1	0.0256	0.5082
2	4	2-4	10	0.0286	0.5559
2	4	2-4	100	0.0381	0.6809
2	4	2-4	1000	0.0429	0.7320

Figure 4C

Colony	Bee Name	Subject	Quantity/ng	Response (mV)	LogResponse+2.1
1	1	1-1	control	0.0476	0.7778
1	1	1-1	1	0.0381	0.6809
1	1	1-1	10	0.0524	0.8192
1	1	1-1	100	0.0476	0.7778
1	1	1-1	1000	0.0476	0.7778
1	1	1-1	10000	0.0476	0.7778
1	1	1-1	100000	0.0619	0.8917
1	2	1-2	control	0.0238	0.4768
1	2	1-2	1	0.0381	0.6809
1	2	1-2	10	0.0405	0.7072
1	2	1-2	100	0.0524	0.8192
1	2	1-2	1000	0.0571	0.8570
1	2	1-2	10000	0.0571	0.8570
1	2	1-2	100000	0.0952	1.0788
1	3	1-3	control	0.0238	0.4768
1	3	1-3	1	0.0190	0.3798
1	3	1-3	10	0.0190	0.3798
1	3	1-3	100	0.0238	0.4768
1	3	1-3	1000	0.0262	0.5181
1	3	1-3	10000	0.0333	0.6229
1	3	1-3	100000	0.0667	0.9239
1	4	1-4	control	0.0381	0.6809
1	4	1-4	1	0.0333	0.6229
1	4	1-4	10	0.0238	0.4768
1	4	1-4	100	0.0214	0.4310
1	4	1-4	1000	0.0238	0.4768
1	4	1-4	10000	0.0238	0.4768
1	4	1-4	100000	0.0619	0.8917
2	1	2-1	control	0.0190	0.3798
2	1	2-1	1	0.0190	0.3798
2	1	2-1	10	0.0200	0.4010
2	1	2-1	100	0.0214	0.4310
2	1	2-1	1000	0.0286	0.5559
2	1	2-1	10000	0.0405	0.7072
2	1	2-1	100000	0.0810	1.0082
2	2	2-2	control	0.0238	0.4768
2	2	2-2	1	0.0262	0.5181
2	2	2-2	10	0.0295	0.5702
2	2	2-2	100	0.0300	0.5771
2	2	2-2	1000	0.0295	0.5702
2	2	2-2	10000	0.0295	0.5702
2	2	2-2	100000	0.0667	0.9239
2	3	2-3	control	0.0238	0.4768
2	3	2-3	1	0.0310	0.5907
2	3	2-3	10	0.0381	0.6809
2	3	2-3	100	0.0381	0.6809
2	3	2-3	1000	0.0429	0.7320
2	3	2-3	10000	0.0476	0.7778
2	3	2-3	100000	0.0833	1.0208
2	4	2-4	control	0.0381	0.6809
2	4	2-4	1	0.0390	0.6916
2	4	2-4	10	0.0452	0.7555
2	4	2-4	100	0.0429	0.7320
2	4	2-4	1000	0.0438	0.7416

2	4	2-4	10000	0.0857	1.0331	2	4	2-4	10000	0.0429	0.7320
2	4	2-4	100000	0.1905	1.3798	2	4	2-4	100000	0.0857	1.0331
3	1	3-1	control	0.0216	0.4350	3	1	3-1	control	0.0190	0.3798
3	1	3-1	1	0.0232	0.4658	3	1	3-1	1	0.0333	0.6229
3	1	3-1	10	0.0262	0.5181	3	1	3-1	10	0.0381	0.6809
3	1	3-1	100	0.0333	0.6229	3	1	3-1	100	0.0390	0.6916
3	1	3-1	1000	0.0381	0.6809	3	1	3-1	1000	0.0390	0.6916
3	1	3-1	10000	0.0952	1.0788	3	1	3-1	10000	0.0476	0.7778
3	1	3-1	100000	0.2857	1.5559	3	1	3-1	100000	0.0810	1.0082
3	2	3-2	control	0.0216	0.4350	3	2	3-2	control	0.0238	0.4768
3	2	3-2	1	0.0208	0.4188	3	2	3-2	1	0.0333	0.6229
3	2	3-2	10	0.0238	0.4768	3	2	3-2	10	0.0429	0.7320
3	2	3-2	100	0.0357	0.6528	3	2	3-2	100	0.0390	0.6916
3	2	3-2	1000	0.0357	0.6528	3	2	3-2	1000	0.0333	0.6229
3	2	3-2	10000	0.1333	1.2249	3	2	3-2	10000	0.0381	0.6809
3	2	3-2	100000	0.2667	1.5260	3	2	3-2	100000	0.0667	0.9239
3	3	3-3	control	0.0359	0.6552	3	3	3-3	control	0.0286	0.5559
3	3	3-3	1	0.0351	0.6455	3	3	3-3	1	0.0371	0.6699
3	3	3-3	10	0.0476	0.7778	3	3	3-3	10	0.0452	0.7555
3	3	3-3	100	0.0524	0.8192	3	3	3-3	100	0.0476	0.7778
3	3	3-3	1000	0.0571	0.8570	3	3	3-3	1000	0.0452	0.7555
3	3	3-3	10000	0.0952	1.0788	3	3	3-3	10000	0.0452	0.7555
3	3	3-3	100000	0.2095	1.4212	3	3	3-3	100000	0.0857	1.0331
3	4	3-4	control	0.0288	0.5589	3	4	3-4	control	0.0190	0.3798
3	4	3-4	1	0.0256	0.5082	3	4	3-4	1	0.0190	0.3798
3	4	3-4	10	0.0286	0.5559	3	4	3-4	10	0.0262	0.5181
3	4	3-4	100	0.0381	0.6809	3	4	3-4	100	0.0286	0.5559
3	4	3-4	1000	0.0381	0.6809	3	4	3-4	1000	0.0286	0.5559
3	4	3-4	10000	0.0714	0.9539	3	4	3-4	10000	0.0286	0.5559
3	4	3-4	100000	0.1476	1.2691	3	4	3-4	100000	0.0476	0.7778

Figure 5 Bioassay of STDs within two hours

Sites	Sample	N of males	Weather
YNAU	control	0	Sunny
YNAU	20 µg OOA	0	Sunny
YNAU	20µg ODA	0	Sunny
YNAU	15 µg OOA+5µg ODA	1	Sunny
YNAU	5 µg OOA+15µg ODA	2	Sunny
YNAU	1 gynes	4	Sunny
YNAU	8.7 µg OOA+11.3 µg ODA	3	Sunny
SWRC1	control	0	Sunny
SWRC1	20 µg OOA	0	Sunny
SWRC1	20µg ODA	0	Sunny
SWRC1	15 µg OOA+5µg ODA	0	Sunny
SWRC1	5 µg OOA+15µg ODA	0	Sunny
SWRC1	1 gynes	3	Sunny
SWRC1	8.7 µg OOA+11.3 µg ODA	5	Sunny
SWRC2	control	0	Sunny
SWRC2	20 µg OOA	0	Sunny
SWRC2	20µg ODA	0	Sunny
SWRC2	15 µg OOA+5µg ODA	0	Sunny
SWRC2	5 µg OOA+15µg ODA	0	Sunny
SWRC2	1 gynes	3	Sunny
SWRC2	8.7 µg OOA+11.3 µg ODA	7	Sunny
